# Membrane Transporters Involved in Iron Trafficking: Physiological and Pathological Aspects

**DOI:** 10.3390/biom13081172

**Published:** 2023-07-27

**Authors:** Andrea Pasquadibisceglie, Maria Carmela Bonaccorsi di Patti, Giovanni Musci, Fabio Polticelli

**Affiliations:** 1 Department of Sciences, University Roma Tre, 00146 Rome, Italy; andrea.pasquadibisceglie@uniroma3.it; 2 Department of Biochemical Sciences ‘A. Rossi Fanelli’, Sapienza University of Rome, 00185 Rome, Italy; mariacarmela.bonaccorsi@uniroma1.it; 3 Department of Biosciences and Territory, University of Molise, 86090 Pesche, Italy; giovanni.musci@unimol.it; 4 National Institute of Nuclear Physics, Roma Tre Section, 00146 Rome, Italy

**Keywords:** iron transport, ferroportin, transferrin, DMT1, ZIP8, ZIP14, mitoferrin, pathogenic mutations

## Abstract

Iron is an essential transition metal for its involvement in several crucial biological functions, the most notable being oxygen storage and transport. Due to its high reactivity and potential toxicity, intracellular and extracellular iron levels must be tightly regulated. This is achieved through transport systems that mediate cellular uptake and efflux both at the level of the plasma membrane and on the membranes of lysosomes, endosomes and mitochondria. Among these transport systems, the key players are ferroportin, the only known transporter mediating iron efflux from cells; DMT1, ZIP8 and ZIP14, which on the contrary, mediate iron influx into the cytoplasm, acting on the plasma membrane and on the membranes of lysosomes and endosomes; and mitoferrin, involved in iron transport into the mitochondria for heme synthesis and Fe-S cluster assembly. The focus of this review is to provide an updated view of the physiological role of these membrane proteins and of the pathologies that arise from defects of these transport systems.

## 1. General Iron Metabolism and Trafficking

Among all metals, iron is probably the one that most exhibits a double face in living organisms: essential for numerous biological functions, from oxygen and electron shuttling to DNA synthesis and repair [1]; harmful because of its ability to monoelectronically reduce oxygen, triggering physiologically defensive or pathologically dangerous oxidative stress cascades [2,3].

In addition to, and in conjunction with, possessing this two-faced behavior, iron is also difficult for cells to handle. In fact, the largely prevalent form in the aerobic environment is the ferric ion Fe^3+^. However, Fe^3+^ salts are very poorly soluble in polar solvents like water. All organisms, therefore, have evolved mechanisms of iron import, export and storage aimed, on the one hand, at accumulating and utilizing the metal in bioavailable forms and, on the other hand, at keeping its oxidoreductive exuberance under control.

Mammals have no regulated pathways of iron excretion, so the amount of metal absorbed with the diet must balance the iron lost by bleeding or by sloughing of epithelial cells. An adult human body contains 3–4 g of iron, and daily losses amount to 1–2 mg. In mammalian cells, iron is mainly distributed in mitochondria (ca. 16 μM), cytosol (ca. 6 μM), nuclei (ca. 7 μM) and lysosomes (ca 16 μM) [4]. Therefore, all cells contain and use iron. However, overall metal homeostasis is governed primarily by very few cellular types: erythrocytes and their precursors, which contain about two-thirds of all the iron in the body; hepatocytes and macrophages, which are reservoir sites for the metal; and enterocytes, which, along with macrophages, provide the gateway for the metal to enter the circulatory system, the blood being the last of the compartments with a key role in the iron game.

Uptake of dietary inorganic iron is due to the presence of DMT1 (divalent metal transporter 1) on the apical side of enterocytes [5]. This transporter handles reduced Fe^2+^ as a substrate, but most dietary iron is in the oxidized Fe^3+^ form. Therefore, iron has to be reduced before it can be absorbed. This is achieved through duodenal cytochrome B (CYBRD1) and possibly other reductases [6].

For iron to cross the basolateral membrane of enterocytes and enter the bloodstream, a dual system is in place, constituted by ferroportin (FPN), the sole known exporter of iron in multicellular organisms, and hephaestin (HEPH), a multicopper ferroxidase required to load the metal onto transferrin, the iron carrier of the blood [7]. Most of the circulating iron, however, does not come from dietary absorption, but rather from metal recycling, with splenic macrophages playing a major role as collectors of iron-rich senescent erythrocytes. Macrophages also use FPN to load Fe^3+^ onto circulating transferrin; in this case, the ferroxidase partner is ceruloplasmin (CP), a paralog of HEPH [8]. It is important to mention that FPN has been recently shown to be highly abundant on red blood cell membrane [9]. Multiple pieces of evidence point to the role of erythroid cells FPN in maintaining systemic iron homeostasis and in protecting red blood cells from oxidative stress [9,10].

Transferrin (TF) is an 80-kDa bilobal glycoprotein synthesized by hepatocytes and consisting of two homologous N- and C-lobes. Each lobe contains a high-affinity (K_d_ ≅ 10^−20^ M) [11] iron-binding site formed by four amino acid ligands: a histidine, an aspartate and two tyrosine residues. Metal binding also requires the presence of a carbonate anion, anchored to a conserved arginine residue, that occupies the two remaining coordination sites. Selective iron delivery to cells in peripheral tissues occurs through the specific interaction of Fe-TF with its receptor TFRC, a type II transmembrane homodimeric glycoprotein. The TF-TFRC complex readily enters cells by clathrin-mediated endocytosis. The interaction between TFRC and TF is dependent on pH; at pH 7.4, TFRC binds to iron-loaded holo-TF but not to iron-free apo-TF. In contrast, at a lower pH in the endosome, TFRC binds to apo-TF but not to holo-TF [12]. In the endosome, acidification allows the release of Fe^3+^, which is reduced to Fe^2+^ by the ferrireductase STEAP3 [13] and poured into the cytoplasm by DMT1 [14], and probably by ZIP14 [15] and by type IV mucolipidosis-associated protein TRPML1 [16]. Iron-free TF remains bound to TFRC in the acidified endosome and is finally recycled, returning to the cell surface where the complex dissociates. Corollary to this mechanism is the fact that only a fraction of transferrin circulates in the blood bound to iron: in fact, the average saturation of the protein is around 30 percent.

A second receptor for TF has been identified about two decades ago (see reference [17] for a review). TFR2 shares structural and functional similarities with TFRC, despite that its regulatory machine appears peculiar. Hereditary hemochromatosis protein (HFE), a protein belonging to the family of major histocompatibility complex class I molecules, competes with TF for TFR2 and may thereby block the binding of Fe-TF to TFR2, preventing internalization of the complex and negatively regulating cellular uptake of TF-bound iron.

**Figure 1:**
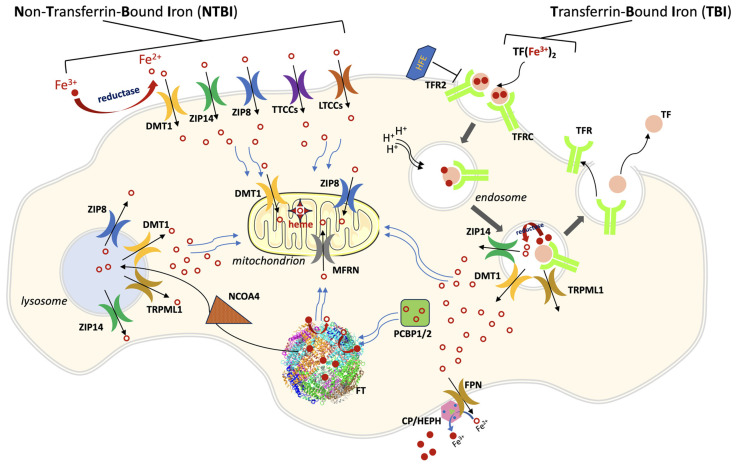
A general scheme of cellular iron traffic. Iron can enter cells both as TBI (transferrin-bound iron) or as NTBI (non-transferrin-bound iron). Iron-loaded transferrin binds to its ubiquitous receptor TFRC (and in some cell types to TFR2) on the cell membrane and the complex is endocytosed. The endosome is acidified, iron is released from TF and poured into cytoplasm through divalent metal transporter 1 (DMT1), and possibly through ZIP14 and type IV mucolipidosis-associated protein TRPML1. The empty complex dissociates and is then recycled outside the cell for a new transport cycle. NTBI circulates in the plasma as Fe^3+^ in low-molecular weight complexes or bound to albumin. After a reduction step, it is taken up as Fe^2+^ through different importers, depending on the cell type: ZIP14, ZIP8, DMT1, LTCCs (L-type Ca^2+^ channels), TTCCs (T-type Ca^2+^ channels). ZIP8 and ZIP14 are also present on lysosomal, mitochondrial, and endosomal membranes. Iron trafficking in the cytosol is mediated by PCBP1/2 (poly(rC) binding protein 1/2), which cargos the metal to ferritin (FT), the iron storage protein, and to other iron-containing enzymes (not shown). In the mitochondrion, the main iron-transport systems are constituted by mitoferrin 1/2 (MFRN) on the inner membrane, and by DMT1 and ZIP8 on the outer membrane. Ferritin iron stores are mobilized through the action of Nuclear Receptor Coactivator 4 (NCOA4), which directs ferritin to the lysosome for degradation. Lysosomes pour iron into cytoplasm through at least four exporters (ZIP8, ZIP14, DMT1 and TRPML1). Cellular iron export is mediated by ferroportin (FPN), which acts in concert with a multicopper ferroxidase (CP, ceruloplasmin or HEPH hephaestin, depending on the cell type).

Several cell types, including neurons, hepatocytes and cardiomyocytes, can uptake iron in the form of the so-called non-transferrin-bound iron (NTBI), a potentially toxic form of the metal that results from pathological excessive entry of iron into plasma, for example in hereditary hemochromatosis and thalassemia major. Under these conditions, the saturation capacity of transferrin is exceeded and iron complexes with a number of low-molecular weight molecules, as well nonspecifically with other plasma proteins. Plasma NTBI is rapidly cleared by most cells through less selective but highly efficient iron transporters, including SLC39A14 (ZIP14 [18]), SLC39A8 (ZIP8 [19]), DMT1 [20], L-type Ca^2+^ channels (LTTCs [21]) and T-type Ca^2+^ channels (TTCCs [22,23]). Comprehensive reviews on NTBI transporters can be found in the literature [24,25]. A large part of the ferrous NTBI is translocated into the mitochondria for the biogenesis of heme and Fe-S clusters. This is mainly allowed by mitoferrin (MFRN), a small transmembrane transporter (~300 residues) located on the inner mitochondrial membrane [26]. A role in mitochondrial iron trafficking has also been shown for ZIP8 [27] and DMT1 [28,29,30]. A general scheme of cellular iron traffic is depicted in Figure 1.

When intracellular iron levels exceed metabolic needs, two mechanisms intervene to prevent the unwanted presence of the metal and its potentially toxic consequences. On the one hand, intracellular levels of ferritin (FT), a multimeric protein composed of 24 subunits that functions as a veritable cage for iron, increase. Over 4000 iron atoms can be sequestered in a redox-inactive form by FT inside its core [31], with the iron chaperones PCBP1 and PCBP2 enhancing the process [32,33]. On the other hand, levels of FPN on the plasma membrane also increase. As expected, when the cell goes into iron deficiency, the opposite mechanisms are triggered: levels of the TF receptor TFRC increase and synthesis of FT and FPN decreases. This is mainly mediated at the translational level by the presence on the corresponding mRNAs of iron-responsive elements (IREs) which govern the rate of protein synthesis [34]. Very recently, a connection between the iron saturation rate of TF and the levels of FPN on the cell membrane in endothelial cells has been reported [35].

Systemic iron levels must also be tightly regulated. Iron overload leads to hemochromatosis, a detrimental condition that affects parenchymal organs including the liver, heart, and pancreas [36]. Iron deficiency leads to anemia, a global health problem with several pathological consequences including cognitive developmental defects in children, poor physical performance, and unfavorable pregnancy outcomes [37]. It has long been known that, under physiological conditions, blood iron levels are remarkably stable, even when dietary intake is highly variable. It is also known that intestinal absorption of iron increases several times under conditions of metal deficiency and decreases when iron is in excess, demonstrating that iron homeostasis is under endocrine control. The key player is in fact hepcidin (HAMP), a small 25-aa peptide hormone synthesized in the liver [38], that acts as an inhibitor of FPN and whose levels are positively or negatively regulated at the transcriptional level depending on physiological conditions. Activation of the immune system leads to iron retention in macrophages and reduced dietary iron absorption as the result of a massive production of several pro-inflammatory cytokines. Among these, interleukin 6 (IL6) is the main stimulator of the hepatic synthesis of hepcidin through STAT3 [39]. Levels of TF saturation and of intracellular iron stores also positively regulate the synthesis of hepcidin. On the other hand, activation of erythropoiesis leads to the synthesis of the erythroid factor erythroferrone (ERFE), which is a negative regulator of hepcidin [39]. As detailed below, the hormone silences FPN by physically interacting with it and decreases its activity by two mechanisms: on the one hand, it binds in the central cavity of the transporter inhibiting the passage of iron [40]; on the other, binding of hepcidin in a specific pocket of FPN induces its internalization and degradation [41].

## 2. Ferroportin

**Figure 2:**
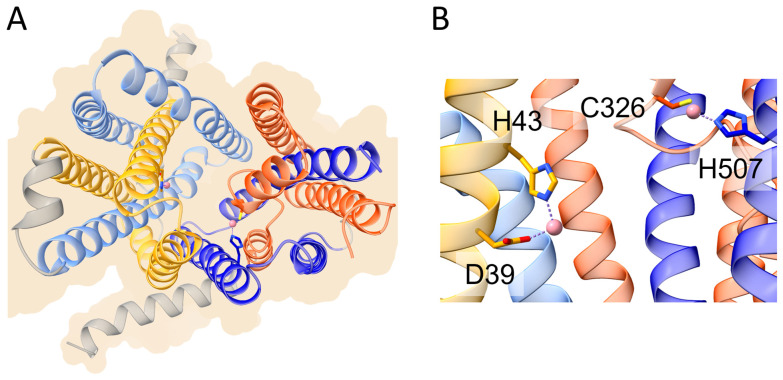
Schematic representation of the overall structure and metal binding sites of human ferroportin (PDB ID 8Dl8 [54]). (**A**) Overall structure of human ferroportin shown in ribbon representation. The helices of the first and second inverted repeats of the N-domain are colored in yellow and cornflower blue, respectively. The helices of the corresponding inverted repeats of the C-domain are colored in orange and blue, respectively (see text for details). (**B**) Metal binding sites on the N-domain (left) and C-domain (right). The cobalt ions mimicking iron are shown as pink spheres. Metal ligands are shown in stick representation.

Ferroportin (FPN) is the only known human iron exporter [42]. Mutations of FPN cause type 4 haemochromatosis, a disease characterized by two different iron accumulation phenotypes [43]. These different phenotypes are the result of loss-of-function mutations, which impair the transport activity, or gain-of-function mutations, affecting hepcidin-mediated FPN internalization and degradation [41]. The first attempts to shed light on FPN structure through molecular modelling techniques revealed that FPN is a member of the Major Facilitator Superfamily (MFS) [44,45,46]. MFS members represent the largest class of secondary active transporters [47,48]. They share a conserved fold, characterized by 12 transmembrane helices forming an N-terminal and a C-terminal domain, each organized into a pair of inverted 3-plus-3 helices repeats, connected by a cytoplasmic loop [48]. In the N-domain, helices 1, 2 and 3 are related to helices 4, 5 and 6 by a 180° rotation around an axis parallel to the membrane bilayer. Similarly, in the C-domain, helices 7, 8, 9 are related to helices 10, 11 and 12 [49] (Figure 2).

**Figure 3:**
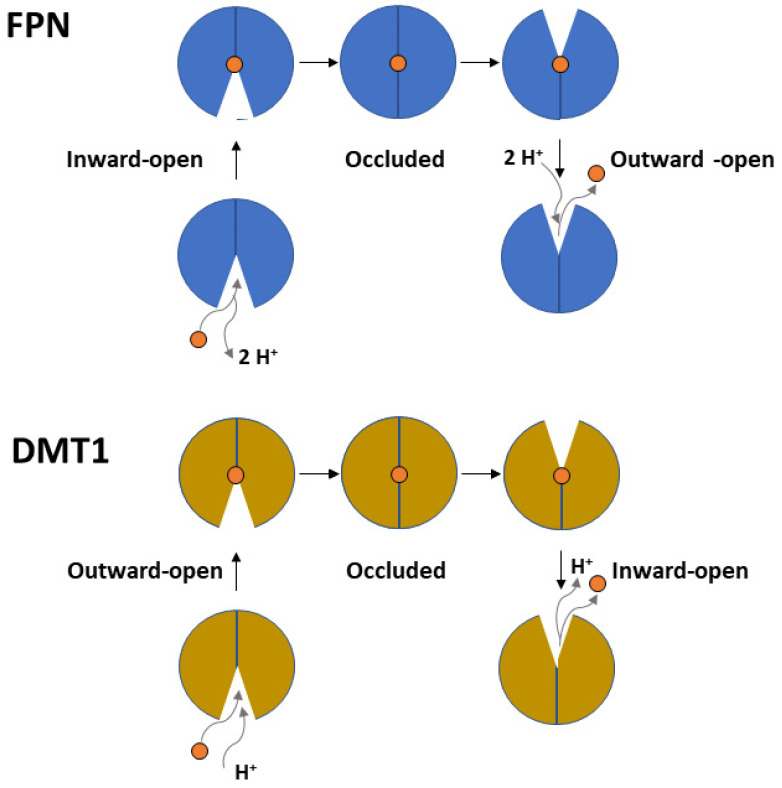
Top panel. Scheme depicting the proton-coupled, electroneutral Fe^2+^ transport mediated by FPN through cycling from an inward-open to an occluded and outward-open conformation. Bottom panel. Similar scheme depicting the proton-coupled-symport of Fe^2+^ mediated by DMT1. See text for details.

The FPN transport cycle occurs through a process known as alternating access mechanism, in which conformational changes between different states (inward-open, occluded, and outward-open) take place. This process relies on the rigid-body relative rotation of the N-terminal and C-terminal domains of FPN, as depicted in Figure 3 [48,49,50]. During this process, the first set of helices (helices 1, 4, 7 and 10) and second set of helices (helices 2, 5, 8 and 11) within each repeat of the protein, which in the inward-open state interact along the cytoplasmic ends, undergo a conformational change and switch their interaction from the cytoplasmic to the extracellular ends of the protein. This alternating access mechanism, driven by the rotation of the N-terminal and C-terminal domains, enables the movement of substrates (such as iron) across the cell membrane.

It has been demonstrated that FPN acts through an electroneutral export mechanism by coupling the export of one Fe^2+^ ion to the import of two H^+^ [51], although other data suggest that FPN may be a Fe^2+^/H^+^ symporter [52]. Structural studies of mammalian FPN evidenced the presence of two metal binding sites [51,53], the first composed by Asp39 and His43, and the second by Cys326 and His507. Recently, structural and functional studies demonstrated that FPN can mediate Ca^2+^ influx through a uniport mechanism and that Asp39 is also involved in binding this metal. In fact, Ca^2+^ is observed to be bound in the vicinity of Asp39 and the Asp39Ala mutation abolishes Ca^2+^ transport [54]. While Fe^2+^ ions inhibit Ca^2+^ transport, the opposite does not hold. However, the physiological significance of Ca^2+^ transport by FPN is still unclear.

Hemochromatosis type 4 is an autosomal dominantly inherited iron overload pathology caused by the mutation of FPN. Two distinct phenotypes are observed, where iron overload affects different organs: mainly reticulo-endothelial macrophages with low transferrin saturation (type 4A) or liver hepatocytes with high transferrin saturation (type 4B) [55,56]. Over 60 missense mutations have been identified in FPN and they have been classified as either loss-of-function or gain-of-function. Loss-of-function mutations cause hemochromatosis type 4A, also named ‘ferroportin disease’, while gain-of-function variants give rise to hemochromatosis type 4B. Loss of function is due to either a direct impact on the capacity of the protein to transport iron or to a folding/subcellular localization defect. Impairment of iron transport can derive from decreased iron binding, such as for the Asp181Val/Asn mutant [57], and/or from the alteration of interactions necessary for the conformational switch required for the translocation of iron across the membrane. An example is the Arg178Gln mutation that disrupts an inter-lobe salt bridge with Asp473, causing destabilization of the outward open state of FPN [58]. Of note, all residues composing motif A (Gly80-Asp84-Arg88) and Asp157, which establish a network of interactions that stabilize the outward-open conformation of FPN, have been found to be mutated in patients, clearly indicating a critical role for conformational transitions in the iron transport cycle [56,59]. Variants Asp157Gly/Tyr/Asn are classified as loss-of-function, while the newly discovered Asp157Ala one appears to lead to a gain-of-function [60], highlighting the difficulty in predicting the effect of mutations. Gain-of-function mutations lead to resistance of FPN to hepcidin, either by the inability to bind the peptide in the central cavity of FPN in the outward conformation or by altering the internalization/ubiquitination process. Interestingly, Cys326Ser/Tyr/Phe and His507Arg mutations, which target residues directly involved in iron binding, are classified as gain-of-function because they also lead to hepcidin resistance, possibly due to the overlap of binding sites for the metal and the peptide. The experimentally determined three-dimensional structures of human FPN have allowed to most mutations to be mapped and a clearer picture of their molecular impact to be obtained. However, full understanding is still lacking given the fact that some mutations are found in regions of the protein that are not resolved, and that structures of FPN are available for the outward-open and occluded states but not for the inward-open conformation. A quite complete, though non-exhaustive list of gain-of-function and loss-of-function mutations with a discussion on their pathogenic role can be found in Tortosa et al., 2017 and references therein [59].

Among gain-of-function mutations, those affecting internalization/ubiquitination of FPN are the most intriguing and are less understood from a molecular point of view. In fact, the details of the process of ubiquitination and internalization have been only partially deciphered. Lysine residue(s) located in the unresolved long intracellular loop connecting the N- and C-terminal domains of FPN are the target for polyubiquitin addition. Evidence suggesting the E3 ubiquitin-ligase Rnf217 [61] and the E1 enzyme UBA6 together with the adaptor protein NDFIP1 [62] are involved in degradation of FPN has been recently reported. The redundancy of pathway components makes it challenging to exactly define which target lysines on FPN and which ubiquitinating enzymes are required for hepcidin-dependent degradation of the transporter. To further complicate matters, FPN has also been reported to be subjected to sumoylation, with the gain-of-function mutation Lys240Glu disrupting this process [63].

Another physiologically relevant aspect that is beginning to be elucidated is the identity of the intracellular donor of ferrous iron to FPN. Recently, it has been determined that PCBP2, originally described as poly(rC) RNA binding protein 2, is in fact a dual-function protein, acting also as an iron chaperone [32]. PCBP2 is a ubiquitously expressed cytosolic protein composed of three KH domains. FPN was demonstrated to interact with the iron-loaded form of PCBP2 only. The iron-dependent interaction was determined to involve the cytoplasmic C-terminal region of FPN and the KH2 domain of PCBP2 [64]. Remarkably, PCBP2 directly links iron uptake to iron export in the cell: the iron chaperone receives the metal from DMT1 (see below) and delivers it to the iron exporter FPN.

## 3. DMT1

**Figure 4:**
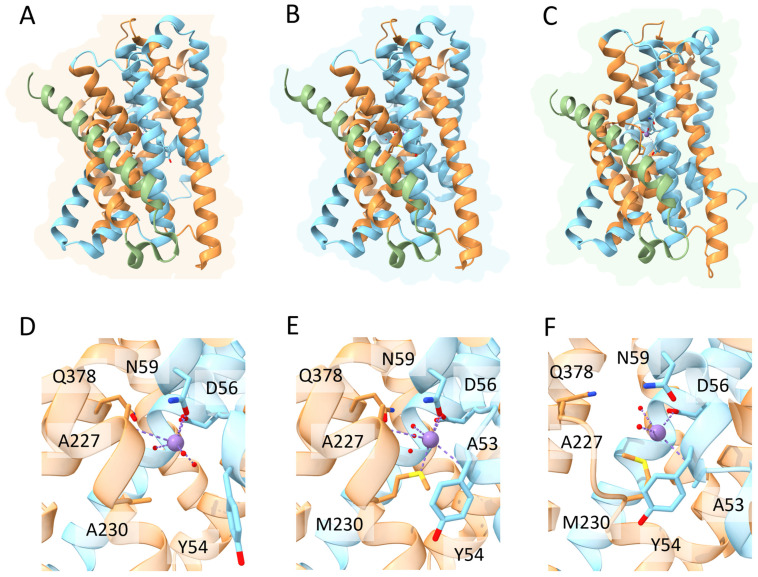
Schematic representation of the relevant conformational states and metal binding sites of *Deinococcus radiodurans* NRAMP. Top panels. Overall structure of the (**A**) inward-open; (**B**) occluded and (**C**) outward open conformational states of the transporter (PDB IDs 8E6I, 8E60 and 8E6N, respectively [73]). The first five helices are colored in gold, the second five helices in cyan and the additional C-terminal helix in green (see text for details). Bottom panels. Detailed view of the Mn^2+^ ion coordination sphere in the (**D**) inward-open; (**E**) occluded and (**F**) outward open conformational states. Mn^2+^ ion is shown as a purple sphere, water molecules as small red spheres and coordinating residues in stick representation.

DMT1 belongs to the ubiquitous solute carrier 11 (SLC11) family of transporters. In vertebrates, DMT1 (SLC11A2) is the primary transporter of Fe^2+^ from the intestinal lumen to the cytoplasm of enterocytes. In addition, DMT1 is also responsible for the transport of Fe^2+^ from the interior of intracellular vesicles to cytoplasm (see Manatschal and Dutzler, 2022 [65] for a recent comprehensive review covering the structural and functional aspects). In both cases, iron transport is coupled to co-transport of H^+^ [66]. Interestingly, DMT1 localization on the outer membrane of mitochondria has also been evidenced [28,29], and a recent study demonstrated that DMT1 is involved in mitochondrial uptake of Fe^2+^ and Mn^2+^ [30]. Indeed, several studies evidenced the rather broad specificity of human DMT1 that can also transport Co^2+^, Mn^2+^, Cd^2+^ and Ni^2+^, although Fe^2+^ is the preferred substrate [67]. From a structural viewpoint, studies on the bacterial orthologs of the DMT1/NRAMP family identified in *Staphylococcus capitis* [68], *Deinococcus radiodurans* [69,70] and *Escherichia coli* [71] revealed a fold first observed in the *Aquifex aeolicus* LeuT amino acid transporter. The main characteristic of this fold is the presence of twelve transmembrane helices (TM), of which the first ten are organized into two domains (TM1–5 and TM6–10) related by a pseudo-two-fold axis in the membrane plane [72] (Figure 4). Interestingly, the central substrate binding site is made up by residues located on TM1 and TM6 in unwound regions [73]. As already described for the MFS members like FPN, the transport cycle involves conformational changes between an outward-open and an inward-open state, through an intermediate occluded state (Figure 3) [65]. Very recently, novel evidence on the metal transport pathway of the members of the DMT1/NRAMP has been obtained through structural analysis of *Deinococcus radiodurans* NRAMP bound to Mn^2+^ in all the three relevant conformational states [73]. The structures clearly highlight how global conformational changes are driven by changes in the coordination sphere of the metal, made possible by the relatively high flexibility of the TM1 and TM6 helices on which metal binding residues are located (Figure 4).

Mutations of DMT1 are very rare and are generally associated to hypochromic microcytic anemia with iron overload. Only 10 patients have been described so far, and distinct missense and splicing mutations have been identified either in the homozygous or compound heterozygous state [74,75]. Not all missense variants have been fully characterized from a biochemical point of view, but mutations appear to affect DMT1 iron transport activity and/or subcellular localization. The homozygous mutation Glu399Asp causes preferential skipping of exon 12 [76]. The Glu399Asp variant appears to be fully functional, but most DMT1 transcripts in the patient lacked exon 12, thus encoding a shorter unstable version of the protein. This mutation, therefore, appears to lead to a reduction in protein quantity rather than to loss of function of the missense variant. The variant Arg416Cys was found in an Italian patient as compound heterozygote with a deletion (delCTT) in intron 4 that caused aberrant splicing of exon 5. In this case, DMT1 Arg416Cys exhibited complete loss of function and abnormal localization [77]. A similar situation was described for a patient with potentially damaging substitution Arg477Trp associated to a splicing defect that caused skipping of exon 4 [78]. Mutation Gly212Val was found as compound heterozygote in association with delVal114 [79] or Asn419Ser [80] or a splice site defect [81]. The impact of the Gly212Val substitution is unclear, as is the deletion of Val114. On the other hand, mutation Asn419Ser caused improper protein trafficking. Gly75Arg is the only other homozygous variant identified so far; the replacement of glycine with arginine was shown to affect protein stability and lead to accumulation of DMT1 in lysosomes [75].

At variance with FPN, where alternative splicing produces two isoforms with an identical coding sequence that differ for the presence or absence of an IRE in the 5’UTR [82], alternative splicing of DMT1 produces multiple isoforms with distinct coding sequences with or without an IRE at the 3’UTR. Isoform 1A is expressed in the duodenum and kidney, and has 29 additional amino acids at N-terminus compared to isoform 1B, which is ubiquitous in endosomal membranes. The isoforms +IRE have 18 amino acids, while the -IRE isoforms have 25 residues at the C-terminus [83]. The presence of the additional sequence at the N-terminus determines the plasma membrane localization of DMT1, while the longer C-terminus contains a recycling endosome signal. Whether there is also an impact on iron transport activity of DMT1 remains to be evaluated.

IRE/IRP post-transcriptional regulation and ubiquitin-dependent degradation are the main mechanisms that regulate DMT1 levels. The ubiquitin E3 ligase WWP2 of the NEDD4 family together with the adaptors NDFIP1/2 is involved in the ubiquitination of DMT1 [84].

Interaction with the iron chaperone PCBP2 is complementary to the situation for FPN [85]. In fact, it has been demonstrated that the KH2 domain of iron-depleted PCBP2 interacts with the N-terminal region of iron-loaded DMT1 [85]. A conformational transition is speculated to take place for the transfer of ferrous iron from the transporter to the chaperone. The N-terminal region of DMT1 interacts specifically with iron-depleted PCBP2; in turn, the C-terminal region of FPN interacts with iron-loaded PCBP2. No obvious similarity is evident between these regions of the two iron transporters, suggesting that the conformation of PCBP2 may be modulated by the presence of iron, to favor specific recognition of the correct partner for distribution of the metal.

## 4. ZIP8 and ZIP14

The SLC39 family (also known as Zrt-, Irt-like Proteins or ZIP) includes membrane proteins that transport metal ions from the extracellular environment, or intracellular organelles, into the cytoplasm. This group of proteins plays a crucial role in the uptake and homeostasis of three essential metal ions: zinc, iron, and manganese [86]. In humans, the ZIP transporter family is composed of fourteen members. These are characterized by the presence of eight transmembrane α-helices (TM), having the N- and C-terminal ends facing the extracellular space, or the vesicular lumen in the case of intracellular ZIPs [86]. The entire protein family is characterized by the presence of six residues “P1-P2-x-x-P3” motif on TM4 and TM5, forming the metal binding site, called binuclear metal center (BMC) [86]. 

Recently, it has been suggested that these transporters could function through an “elevator-type” mechanism, where one domain (TM1, TM4, TM5, and TM6) moves with respect to the other (TM2, TM3, TM7, and TM8) along the axis perpendicular to the membrane plane [87,88]. This leads to a 7 Å displacement of the metal binding sites and a rearrangement of the residues forming the extracellular and intracellular hydrophobic plugs, alternately. These latter block access to the binding site and prevent the substrate from diffusing backward. Depending on the conformational state, the disruption of the hydrophobic plugs allows the interaction of one or more gating residues with the translocated substrate. The extracellular gating residues are responsible for gating the substrate towards the BMC, while the intracellular gating residues mediate the release of the substrate towards the cytosol [88].

The ZIP transporters are subdivided into four subfamilies: subfamily I, subfamily II, GufA and the LIV-1 subfamily, also called LZT (LIV1-subfamily of ZIP zinc Transporters) [89]. The LIV-1 subfamily is characterized by the presence of an extracellular domain (ECD), which is subdivided in a helix rich domain (HRD) and a Pro-Ala-Leu (PAL) motif-containing domain (PCD), likely involved in the dimerization of these transporters [89]. 

Two members of the LIV-1 subfamily, ZIP8 (SLC39A8) and ZIP14 (SLC39A14), have been observed to be able to translocate iron [18,19]. Both of them lack the HRD domain, but retain a PCD-like domain able to mediate dimerization [89]. ZIP8 and ZIP14 have been shown to translocate several important divalent metals (e.g., Zn^2+^, Fe^2+^ and Mn^2+^) [90,91]. Although they share a high sequence similarity, they have a different tissue distribution, expression and localization [92]. Indeed, ZIP8 is more abundant in lungs and testes, while ZIP14 is highly expressed in the liver and duodenum [93]. Regarding the subcellular localization, ZIP8 was reported to be located at the plasma membrane [27] and at lysosomes membranes [94], but there is also evidence of ZIP8 localization in mitochondria [27]. Similarly, ZIP14 was detected at the cell surface and at early endosomes and lysosomes membranes [15]. In this regard, ZIP14 is suggested to mediate both NTBI and TBI transport [95] (see Figure 1). This is also supported by the fact that ZIP14 is able to mediate the transport of iron at pH 6.5, at which, in the endosome, more than half of iron-transferrin complexes are dissociated [96]. 

An in vivo study using an inducible ZIP8 KO mouse model showed an increased presence of splenic iron, mainly located in the red pulp macrophages [97]. This suggests that ZIP8 could be involved in iron recycling from red blood cells. However, minor or no effects on haematological parameters were observed, indicating the presence of a redundant intracellular iron translocation system. Moreover, ZIP8 could be also crucial for iron uptake in embryonic and foetal hematopoietic organs, including yolk sac and foetal liver [98]. 

**Figure 5:**
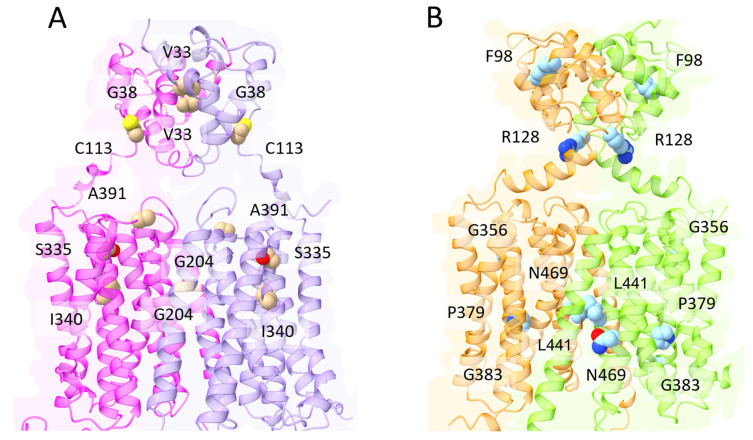
Structural models of the ZIP8 (**A**) and ZIP14 (**B**) dimers. The four monomers are represented in ribbon representation and colored in magenta and violet, in case of ZIP8, and in orange and green, in case of ZIP14. The residues involved in pathogenic mutations described in the text are represented as van der Waals spheres and colored in tan (ZIP8) and in cyan (ZIP14). The two dimers have been obtained with AlphaFold2-mutlimer v2.3.0 [115].

ZIP8 homozygous or heterozygous mutations have been found to cause the Congenital Disorder of Glycosylation Type IIn (CDG2N) [99,100]. CDG2N is an autosomal recessive disorder, characterized by a delayed psychomotor development including symptoms such as: hypotonia, short stature, seizures, visual impairment and cerebellar atrophy. The patients displayed decreased levels of Mn^2+^ and Zn^2+^ in blood, but high in urine, indicating renal wasting and a loss of function of the transporter [99,100]. The Gly38Arg substitution is located in the ECD domain, and the presence of a bulky basic residue in an area composed of small hydrophobic amino acids could affect the folding of the domain and the dimerization process (Figure 5A) [99]. In a second study, this substitution was found in conjunction with the Ile340Asn mutation [100]. This residue is part of the hypothetical hydrophobic plug that is formed in the inward-facing conformation [88]. Therefore, the presence of a polar residue in that region could impair ZIP8 activity. In the same study, a second patient with CDG2N was found with a paternal allele carrying a Val33Met and a Ser335Thr substitution, and a maternal allele with a Gly204Cys substitution [100]. Val33 is located in the ECD domain, and the effect of its substitution with Met (higher steric hinderance) could be similar to that of the Gly38Arg mutation, causing misfolding of the dimerization domain (Figure 5A). Ser335 is likely part of the extracellular gating motif, and its substitution with a Thr residue, although apparently conservative, could perturb the initial recognition of the translocated substrate (Figure 5A) [88]. Gly204Cys is located at the interface with the bilayer membrane lipids (Figure 5A), and it is not clear whether it could be detrimental for the protein function [100].

A screening of 91,713 functional SNP loci in coding regions in 10,523 individuals with inflammatory bowel disease and in 5726 controls, identified an association between Crohn’s disease and a polymorphism in the ZIP8 gene (Ala391Thr) [101]. The authors of this study also discussed the possibility that the association between the ZIP8-Ala391Thr variant and other distinct phenotypes including obesity, lipid levels, blood pressure and schizophrenia could depend on the shift in the gut microbiome composition [101]. Ala391 is located on the loop linking H6 to H7, facing the extracellular space or endosome/lysosome lumen (Figure 5A), but its role in the protein function is not clear, although it has been suggested that its mutation could induce a structural change [102].

ZIP8 Cys113Ser mutant has also been associated with a Leigh-like mitochondrial disorder, where the patients had developmental delay, dystonia, seizures and failure to thrive [103]. This residue is located in the ECD, facing another Cys residue (Cys74) likely forming a disulphide bond (Figure 5A). However, a recent study reported that the ZIP8 mutants Gly38Arg, Gly204Cys and Ser335Thr showed reduced Se uptake, whereas the Cys113Ser variant had a Se transport ability comparable to that of the wild type [104]. It should be mentioned that Choi and colleagues demonstrated that all the ZIP8 pathogenic-mutants caused a mislocalization of the protein to the ER [105]. A preprint study has been recently published reporting an engineered quadruple ZIP8 mutant, in which changing four residues led to an increased Zn^2+^ translocation over Cd^2+^, Fe^2+^ and Mn^2+^ [91]. Intriguingly, two of these residues, Gln180 and Glu343, lie in proximity of the above-mentioned residues Ser335 and Ile340, supporting the notion that mutations in this region could affect both the substrate specificity and the translocation process.

Mice models of ZIP14 KO were resistant to iron overload [106]. Jenkitkasemwong and colleagues indicated ZIP14 as one of the key players in the NTBI uptake route into hepatocytes and pancreatic acinar cells during iron overload, as in the case of hereditary hemochromatosis [107]. In 2018, the same research group demonstrated that ZIP14 is also required for Mn^2+^ uptake in liver and pancreas [108]. In fact, ZIP14 loss leads to Mn accumulation in the brain and in other extrahepatic tissues. However, tissue-specific ZIP14 KO proved that only the intestinal ZIP14 is critical for the systemic homeostasis of Mn [108].

ZIP14 mutations have been linked to two inherited diseases: Hypermanganesemia with Dystonia 2 (HMNDYT2) [109] and hyperostosis cranialis interna [110].

The former is characterized by childhood-onset parkinsonism–dystonia with neurodegenerative features, caused by an increased Mn deposition in the brain [109]. Several missense mutations have been shown to cause HMNDYT2. In particular, Phe98Val, Gly383Arg and Asn469Lys mutations compromised Mn^2+^ translocation activity, without affecting the protein expression or localization [109]. In this regard, Phe98 is located in the ECD, in a region where hydrophobic residues are tightly packed (Figure 5B). The substitution with a smaller residue could affect the correct folding of the domain. Gly383 is located in the intracellular gating area, among many acidic residues (e.g., Asp443) (Figure 5). The presence of a Lys in this position could disrupt the network of electrostatic interactions, considering also that the presence of a positively charged sidechain could interfere with the translocation of the divalent cation. The third mutation, Asn469Lys, is located in TM8, at the interface with the bilayer lipid membrane or with the other monomer of the hypothetical dimer (Figure 5B). In both cases, the residue is surrounded by hydrophobic moieties. In the case of the dimer, Asn469 is predicted to interact with Tyr232. In both cases, a Lys residue in this position could be detrimental for the correct folding and functioning of the protein. Three more mutations have been found associated with HMNDYT2 (i.e., Arg128Trp [111], Pro379Leu [112], Gly356Ser [113]), but there are no functional studies analysing the effect of these mutations. From a structural viewpoint Arg128Trp could affect the proper folding of the dimeric ECD, while Pro379Leu and Gly356Ser are located at transmembrane helices interfaces and the presence of different residues could affect the helices packing and flexibility (Figure 5B). 

Hyperostosis cranialis interna is characterized by an endosteal hyperostosis and osteosclerosis of the calvaria and the skull base [114]. One patient was found to have a missense heterozygous mutation in the ZIP14 gene. The substitution Leu441Arg occurs in a region at the protein-membrane interface, in the case of a monomeric protein, or at the protein-protein interface, in the case of a dimeric one (Figure 5B). In both cases, the presence of a positively charged residue is likely to interfere with the correct tertiary and quaternary structure of the protein [110].

## 5. Mitoferrin

The protein responsible for iron transport in mitochondria is called mitoferrin (MFRN). In humans, two paralogs are present, MFRN1 (SLC25A37) and MFRN2 (SLC25A28), which belong to the Mitochondrial Carrier Family (MCF; SLC25) [26]. While MFRN1 is mainly expressed in erythroid tissue, with minimal expression in other cells, MFRN2 is expressed ubiquitously [116].

These proteins consist of three repeated homologous domains, where each domain is formed by two TM helices connected by a shorter amphipathic helix facing the matrix. All three domains are characterized by a conserved signature motif Px[DE]xx[KR], located on the odd-numbered TM helices, and a less conserved motif [FY][DE]XX[KR], located on the even-numbered helices [117,118]. While the charged residues present in the former motif are responsible for the formation of the matrix salt bridge network, the charged residues of the latter form the cytoplasmic salt bridge network. These two networks are formed alternately; in particular, when the matrix network residues interact, the transporter matrix side is closed and the cavity is opened towards the intermembrane space, while when the cytoplasmic network is formed, the transport cytoplasmic side is closed, and the cavity is opened towards the mitochondrial matrix [117]. The SLC25 members also share a common substrate binding site, consisting of conserved residues known as contact points (CPs), located on the three even-numbered TM helices [119].

MFRNs were first identified in yeast [120] and then biochemically characterized as iron transporters [121]. Indeed, biochemical assays showed that MFRN1 is able to transport manganese, cobalt, copper and zinc, and that iron transport does not need a chelating agent [121]. Interestingly, the ATP-binding cassette transporter ABCB10 was found to bind directly to the MFRN1 transporter, increasing its half-life [122]. 

**Figure 6:**
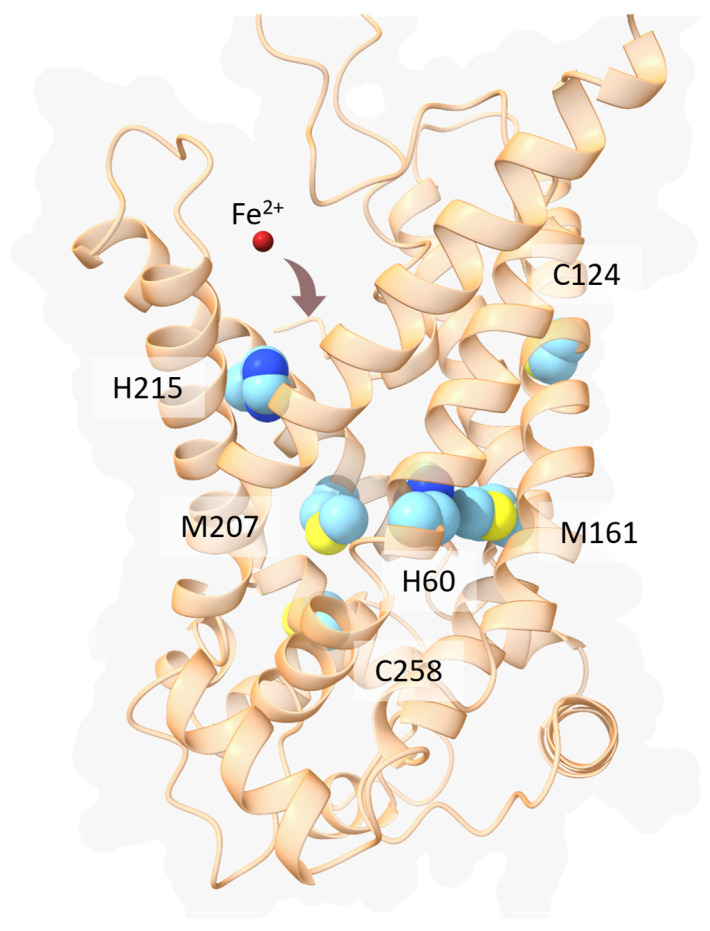
Structural model of the human MFRN1. The protein is in ribbon representation, while the hypothetical iron coordinating residues are shown in sticks and colored in cyan. The ferrous ion is represented as a red sphere. The human MFRN1 structural model was retrieved from the AlphaFold Protein Structure Database [124].

Although the molecular mechanism is still unknown, three conserved His residues could be involved in the translocation of the iron ion [123]. Indeed, a residue, orthologous to one of these His, was found to be critical for the function of a homologous mitoferrin (His48 in the yeast mitochondrial RNA splicing 3 (Mrs3), His55 in *Oreochromis niloticus* MFRN) [121]. Indeed, mutational analyses of *O. niloticus* MFRN suggested that the residues that have the highest effect on the transport activity are His55, Cys119, Met156, Met202, His210 and Cys253 [121]. In human MFRN1, these residues correspond to His60, Cys124, Met161, Met207, His215 and Cys258 (Figure 6).

Of note, His60, Met161 and Met207 are located in correspondence of the canonical central binding site [119]. Since Cys214 and Cys258 are oriented towards the lipid membrane interface, it is unlikely that these residues would be able to interact with the translocated iron ion, at least in the c-state conformation.

*O. niloticus* MFRN1 showed to be sensitive to the truncation of both its C and N termini. The removal of 18 residues from the N terminus or 10 residues from the C terminus resulted in a significant decrease in its ability to transport iron [121]. Furthermore, its ability to transport various first-row transition metal ions is similar to other metal transporters like ZIP14 [125], FPN [126] and DMT1 [67]. 

Given their role, both MFRN1 and MFRN2 are often found to be associated with pathological conditions [26]. A zebrafish mutant for MFRN *frascati* (*frs*) displayed severe hypochromic anaemia and erythroid maturation arrest owing to defects in mitochondrial iron uptake [116]. Although MFRN1 is poorly expressed in non-erythroid tissues, deletion of MFRN1 in hepatocytes during increased porphyrin synthesis led to reduced protoporphyrin IX to heme conversion, causing protoporphyria, cholestasis and bridging cirrhosis [127]. 

High levels of an MFRN1 isoform have been observed in refractory anemia with ring sideroblasts and with marked thrombocytosis (RARS/-T) patients with a SF3B1 mutation [128]. However, this isoform (Ensembl transcript id: ENST00000518881) does not seem to be associated with a protein product. Furthermore, major depressive disorder patients displayed decreased levels of MFRN1 in both the hippocampus and peripheral blood [129]. This is in agreement with MFRN1 KO mice models, which display reduced neuronal energy metabolism and impaired hippocampus-dependent memory [130]. In drosophila models for Friedreich ataxia (FRDA), the MFRN1 homologue was found to be overexpressed, and the relative suppression was able to recover the normal phenotype, although reducing the life span of the organism [131]. In fact, the downregulation of MFRN1 could affect both iron metabolism and reactive oxygen species balance, as shown in a *C. elegans* model for Alzheimer’s disease [132].

As already mentioned, MFRN2 is expressed ubiquitously, but it is unable to compensate MFRN1 deletion, particularly in the case of high heme synthesis [133]. A role for MFRN2 in spermatogenesis was hypothesized following experimental observation of MFRN2-knockout mice with a reduced sperm number and motility [134]. A decreased spermatogenesis was also reported in a drosophila model knockout for the mitoferrin gene *dmfrn* [135]. MFRN2 was found to be overexpressed in aortic endothelial cells of atherosclerosis mice models, while its relative knockdown led to reduced mitochondrial iron overload [136]. High MFRN2 levels were also detected in humans with Huntington’s disease and mice models of this pathology, inversely correlating with expression of frataxin, the enzyme responsible for iron-sulfur clusters assembly [137]. A similar correlation has also been observed in FRDA, where the increased expression of MFRN1 was concomitant with frataxin loss of function [131]. It should be noted that both proteins have been largely associated with cancer progression and resistance, in particular the radioresistance of some tumor types [26,138,139].

## 6. Conclusions

Our knowledge of the iron trafficking processes has been significantly enriched in the last fifteen years with novel key players discovered, and details clarified about their regulation and intermolecular interactions in physiological and pathological states. However, as common in biology, this progress is paralleled by new open issues that need to be addressed. We still lack high resolution, complete structures of fundamental transporters involved in iron trafficking, essential to understand the molecular basis of transport defects caused by pathogenic mutations. As a matter of fact, no structure is available for human DMT1, ZIP8, ZIP14 and mitoferrins, while only partial structures in one conformational state are available for human ferroportin. Further, physiologically relevant details, such as the molecular bases of the interaction between PCBP2 and ferroportin and its regulation, as well as the reasons for the apparent, partial functional redundancy of some of the iron transporters (e.g., DMT1 and ZIPs) are still unclear. Filling these gaps is the challenge to be faced in the future by the scientific community working on iron metabolism.
